# When decisions of others matter to me: an electrophysiological analysis

**DOI:** 10.1186/1471-2202-11-86

**Published:** 2010-07-29

**Authors:** Josep Marco-Pallarés, Ulrike M Krämer, Saskia Strehl, Andrea Schröder, Thomas F Münte

**Affiliations:** 1 Department of Basic Psychology, University of Barcelona, Vall d'Hebron 171. Barcelona, 08035, Spain; 2 Institut d'Investigacions Biomèdiques de Bellvitge (IDIBELL), Feixa Llarga s.n., L'Hospitalet de Llobregat, 08907, Spain; 3 Dept. of Neuropsychology, Otto von Guericke University, Universitätsplatz 2, Gebäude 24, Magdeburg, 39106, Germany; 4 Dept of Neurology, University of Lübeck, Ratzeburger Allee 160, Lübeck, 23538, Germany

## Abstract

**Background:**

Actions of others may have immediate consequences for oneself. We probed the neural responses associated with the observation of another person's action using event-related potentials in a modified gambling task. In this task a "performer" bet either a higher or lower number and could win or lose this amount. Three different groups of "observers" were also studied. The first (neutral) group simply observed the performer's action, which had no consequences for the observers. In the second (parallel) group, wins/losses of the performer were paralleled by similar wins and losses by the observer. In the third (reverse) group, wins of the performer led to a loss of the observer and vice versa.

**Results:**

ERPs of the performers showed a mediofrontal feedback related negativity (FRN) to losses. The neutral and parallel observer groups did similarly show an FRN response to the performer's losses with a topography indistinguishable from that seen in the performers. In the reverse group, however, the FRN occurred for wins of the performer which translated to losses for the observer.

**Conclusions:**

Taking into account previous experiments, we suggest that the FRN response in observers is driven by two evaluative processes (a) related to the benefit/loss for oneself and (b) related to the benefit/loss of another person.

## Background

In everyday life, situations are abundant in which the actions of one person have consequences for another individual. These can range from banal (somebody losing a coin which I can pick up) to life-changing (parents choosing the husband for their daughter). Obviously, actions and their consequences for another person can elicit a whole range of psychological and neural responses in an observer, ranging from the automatic engagement of the mirror neuron system [1-3] to emotional/empathic reactions [4,5].

Situations may roughly be classified into three different classes: First, an action by another person (henceforth: performer) may lead to direct consequences to an observer in that the observer gains when the performer gains and the observer loses when the performer loses. Second, there might be an inverse relationship between the consequences of an action for the performer and the observer, i.e. the observer loses when the performer wins and vice versa. Third, an action of the performer might be of no immediate consequence to the observer. In this last situation the observer might nevertheless engage in mentalizing in order to learn from the other person's actions (c.f. the vast literature on model learning, e.g [6]).

In the current investigation we set out to directly compare these three types of situations, which we will term parallel, reverse, and neutral, using event-related potentials in normal human participants. To make the consequences of the performer's action clear and to simplify the experiment, performers engaged in a gambling task in which actions resulted in smaller and larger monetary gains and losses for the performer and observer.

In order to study the above mentioned processes we draw on previous results addressing the neural events associated with action monitoring and reward processing. Electrophysiologically, two phenomena have been in the focus of this research: in choice reaction time tasks such as the Eriksen flanker task action errors lead to a phasic negativity, the error-related negativity (ERN) which emerges time-locked to the response and has a maximum over the frontocentral midline scalp [7,8]. The ERN is thus elicited in cases in which the performer registers an action error. In other experimental situations critical information about the quality of the performance is given by feedback. Negative feedback has been shown to elicit an electrophysiological response similar to the ERN, the feedback-related negativity (FRN, sometimes also dubbed mediofrontal negativity, MFN, [9,10]). This response is seen between 250 and 400 ms, has a mediofrontal maximum and its main source has been ascribed to the anterior cingulate cortex [9], although additional sources have been found in the posterior cingulate cortex [11,12] and right superior frontal cortex [12].

In addition to experimental situations, in which negative feedback stimuli provide critical learning information [11,13] the FRN has also been found in gambling paradigms whenever the participant incurred a monetary loss [9,14,15]. Critically, in these paradigms the participants are given a choice between a larger and a smaller number on which they bet in a lottery-like gamble resulting in either a gain or a loss of a sum of money corresponding to the chosen number. According to Gehring et al. [9] the process underlying the FRN may be involved in quickly determining the motivational impact of ongoing events. Because in the original Gehring and Willoughby study events were included in which a small loss was the most advantageous outcome of a trial (because the other response option would have resulted in a greater loss), it appears that the FRN responds to the gain/loss status of an event as opposed to whether or not the choice was erroneous or disadvantageous. In other studies, a strong influence of context on the FRN elicited by losses and negative events has been reported [16].

In recent years, first reports have emerged on the neural events accompanying the observation of errors [17] or action consequences [18] of others. Van Schie et al. [17] found that when participants observed an erroneous action of another person a mediofrontal negativity akin the ERN emerged in the observer's ERP thus suggesting that similar neural mechanisms are involved in monitoring one's own actions and the actions of others. Yu and Zhou [18] recorded brain potentials in a gambling task and found a negativity to losses when the participant observed the feedback given according to another person's action. In a related study, Fukuhima and Hiraki [19] compared neural activity to one's own and another person's monetary gain or loss in a competitive two-person gamble. Importantly, in this situation one's monetary gain resulted in the other's loss. These authors found a gender effect, in that women but not men showed an FRN to the other person's loss (and thus their own gain). This was interpreted as indicating an emotional empathic response of the women to the loss of another person. A similar finding in a win situation during an aggressive social exchange has been reported by our group in a subset of participants [20]. Itagaki and Katayama [21] conducted a experiment in which an observer could gain or lose the same amount of money as another (virtual) participant that was supposedly playing a gambling task (cooperative situation) or gain (lose) when the participant lost (gained) in an antagonistic situation. Results showed that the FRN appeared in those conditions in which the observer lost some amount of money regardless of the consequences for the performer. Therefore, Itagaki and Katayama [21] proposed that the observer's FRN reflected the evaluation of the feedback based on the consequences for the observer rather than the performer.

Building on these earlier observations, we set out for the first time to compare the four different conditions, i.e. performer, observer parallel, observer reverse, and observer neutral, in a gambling task. Importantly, rather than employing virtual performers as in some previous studies, performer and observer were placed next to each other in the same recording chamber and ERPs were obtained from both participants. We expected the performer's ERPs to be characterized by an FRN for losses compared to the gains. For the observer-ERPs, we expected that in the parallel condition a similar FRN should be seen for losses which was expected to be greater than in the neutral condition. For the reverse condition, we expected the ERP to be driven by the observer's loss and hence expected a more negative ERP for trials in which the performer won.

## Results

### Behavioral results

On average performers selected the 25 more often (55 ± 8% of choices). The mean gain of the performers was 0.3 ± 2.5 euros. The counting performance of the observer was virtually perfect, i.e. only occasionally counts of the observers differed from the actual number by 1 or 2.

### Event-related potentials

**Figure 1:**
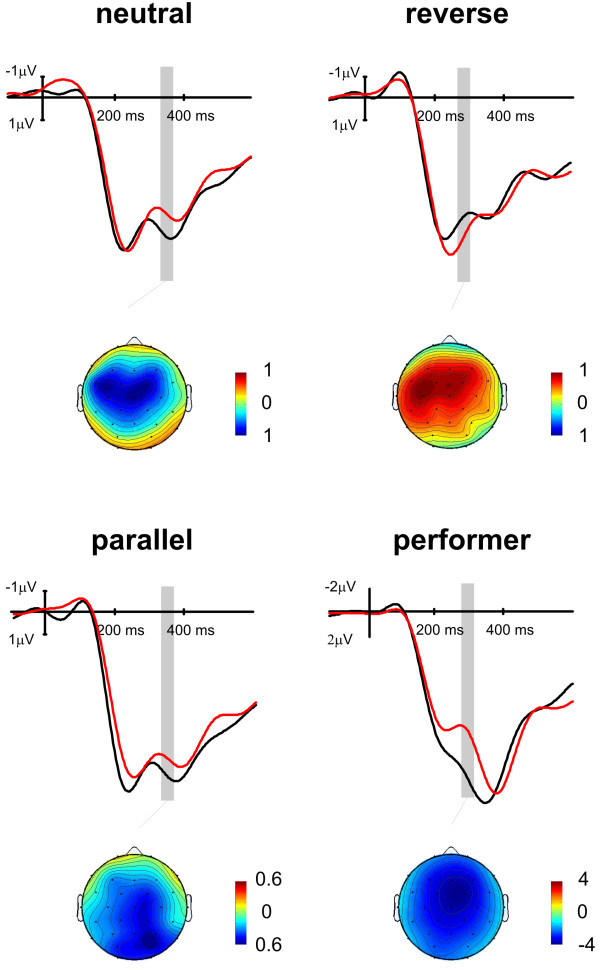
**Event Related Potentials associated to the feedback stimuli**. Event Related Potentials at the Cz electrode associated to the feedback stimuli indicating wins or losses for the performers and the three different observer conditions. Black lines indicate trials in which the performer won (averaged across 25 and 5 cent conditions), whereas red lines indicate the responses associated performer's losses. Note that the observers in the neutral and parallel conditions and the performers showed an increased negativity for performer's losses in the 200-400 ms range. The scalp topographies (isovoltage maps of the loss minus gain difference wave, relative scaling, blue indicating negative, red indicating positive voltages) showed a similar mediofrontal maximum in these three groups. By contrast, observers in the reverse condition showed a more negative response for the performer's gains which translated to losses of the observer. Again, the scalp topography of the difference had a mediofrontal maximum.

Figure 1 shows the event related-potentials to the gains and losses at Cz for the performers and observers (separately for the three different conditions). The performers showed a pronounced positivity for the gains upon which a negativity (FRN) was superimposed for the losses. The gain minus loss difference was maximal at 280 ms. A main effect of valence was present (mean amplitude 260 to 300 ms; F(1,47) = 44.6, P < 0.001). This effect showed a midline frontocentral maximum (electrode × valence (F(28,1316) = 20.1, P < 0.001). In addition, we found a magnitude effect (F(1,47) = 16.5, P < 0.001), showing that the maximum trials showed a greater amplitude than the minimum trials.

Observers in the neutral condition presented a significant negativity associated to losses between 330 and 370 ms (valence F(1,15) = 5.7, P < 0.05, see Table 1) which had a similar distribution as that found for losses in the performers. There was neither a magnitude main effect in the neutral condition, nor an interaction between valence and magnitude.

**Table 1:** Statistical Results

	Performer	Parallel	Reverse	Neutral
**Valence^1^**	44.6***	2.2	12.5**	5.7*

**Magnitude^1^**	16.5***	0.1	1.5	0.4

**Elec^2^**	47.1***	10.1***	11.2***	8.4***

**V × M^1^**	5.1*	9.5**	4.0+	0.5

**V × electrode^2^**	20.0**	1.2	2.7*	2.13

**M × electrode^2^**	2.6***	0.9	0.5	0.8

**V × M × E^2^**	1.9**	3.0*	0.8	0.7

+ *P *< 0.1; * *P *< 0.05, ** *P *< 0.01, *** *P *< 0.001

**^1 ^**degrees of freedom: performer = 1,47; parallel, reverse, neutral = 1,15

**^2 ^**degrees of freedom: performer = 28,1316; parallel, reverse, neutral = 28,420

**Figure 2:**
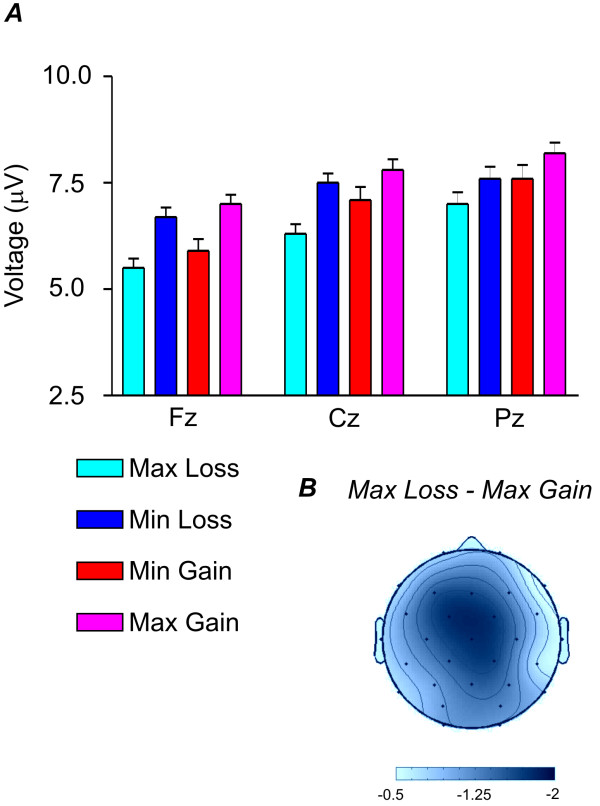
**Observers in the parallel condition**. A. Voltage values of the four different conditions (max gain, min gain, max loss, min loss) for the observers in the parallel condition in the 340-370 ms time range for the three midline electrodes (Fz, Cz, Pz). B. Scalp voltage distribution of the max loss minus max gain condition (relative scaling).

In the parallel condition, again a more negative waveform was found for the losses. We found a significant interaction between magnitude and valence between 260 and 300 ms (F(1,15) = 9.5, P < 0.01), a significant valence × magnitude × electrode interaction in the same time range (F(28,420) = 3.0, P < 0.05, see Table 1), but no significant valence main effect. Figure 2A shows the amplitudes for all trial types for the midline electrodes. It can be seen that there was a greater difference between maximum gains and losses than between minimum wins and losses. When maximum loss and maximum gain were compared, a significant difference was seen (F(1,15) = 10.3, P < 0.01) which showed a frontocentral distribution (see Figure 2B). This differential distribution of the valence effect across midline electrodes was reflected by a marginally significant valence × electrode interaction (F(2,30) = 2.5, P = 0.1). In contrast there were no significant differences between minimum gain and minimum loss (F(1,15) = 1.0, n.s.).

**Figure 3:**
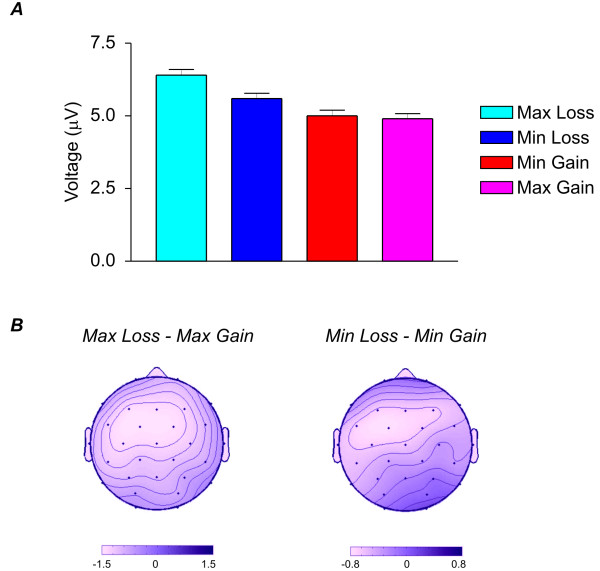
**Observers in the reverse condition**. A. Mean voltage values of the three midline electrodes (Fz, Cz, Pz) of the four different conditions (max gain, min gain, max loss, min loss) for the observers in the reverse condition in the 250-290 ms interval. Note that, as in the rest of the paper, loss and gain refers to the performer. Hence the pattern is opposite to the one shown in Figure 2A, because loss refers to a gain in the observer and vice-versa. B. Scalp voltage distribution of the max loss minus max gain condition (left) and the min loss minus min gain (right). Note the inversion of the effects on the scalp (in this condition loss minus gain in the performer corresponds to gain minus loss in the observer) and the difference in the scales in the two condition (maximum condition: - 1.5 to 1.5 μV; minimum condition: -0.8 to 0.8 μV).

Expectedly, the ERPs for the reverse condition showed an inverse pattern compared to the neutral and parallel conditions in the observers. Here, a more negative ERP could be observed in trials in which the performer won and the observer lost. The difference was maximal at 270 ms. Between 250 and 290 ms a significant valence main effect (F(1,15) = 12.5, P < 0.001), a valence × electrode interaction (F(28,420) = 2.7, P < 0.05) and a marginally significant valence × magnitude interaction (valence F(1,15) = 4.0, P < 0.1) were observed (see Table 1). The difference between maximum gain and maximum loss at midline electrodes (F(1,15) = 19.8, P < 0.001; see Figure 3) was greater than between minimum gain and minimum loss trials (F(1,15) = 2.9, P = 0.1).

## Discussion

Previous research on the effects of feedback about gains or losses in gambling tasks has focused on the neural effects of this information in the performer. It has been concluded that the FRN response seen for losses is reflecting the activity of a performance monitoring network that is engaged in order to optimize future behavior.

The present experiment extends this work in that we have studied the neural responses of persons who observe actions rather than performing themselves. Two important results were obtained: First, we demonstrate that observers show similar brain responses as performers, even if (as in the neutral condition of the present experiment) there is no real consequence of the performer's behavior for the observer. On the other hand, the effects seen in the observer are not just a simple mirror of the performer's brain responses, as in the inverse condition (gains of the performer lead to losses for the observer and vice versa) the brain responses of the performer and observer were similarly reversed, i.e. a more negative waveform was seen in the observer for gains of the performer.

Few previous studies have investigated brain activity in participants observing the outcome of another person's action but each of these studies only investigated a subset of the present conditions and thus no clear picture emerged. Yu and Zhou [18] required their participants to perform a gambling task and observe the performance of another person on alternate trials. The instruction for the observation trials was to learn from the observation how to maximize one's own gain. A similar Feedback Related Negativity was found for the observation and performance condition. This was interpreted to suggest that the observation and performance FRN is the index of a teaching signal, and that the neural processes underlying learning-bydoing are similar to those involved in learning-by-observation. By contrast, in the current experimental paradigm, observers in the neutral condition were neither instructed to learn from the actions of others, nor needed to do so because they were never required to perform themselves during the entire experiment. In spite of the fact that they were simply required to count the occurrences of a certain outcome in any given run, their FRN response was highly similar to that of the performers and that of the observers in the parallel condition. We therefore conclude that an interpretation of the observer's FRN as a reflection of a learning signal is problematic. It is still possible that the observer's FRN in the neutral condition reflects the automatic engagement of a learning mechanism, however, which is called into play whenever we observe other person's decisions and their consequences much like the mirror neurons for motor acts.

Another interpretation is that the FRN in the observers in the neutral condition may reflect an empathic process that occurs even in the absence of an implication of the watched person's action for the observer. Previously, Fukuhima and Hiraki [19] have proposed a similar interpretation for their study in which gender-matched pairs of participants who were acquainted with each other engaged in a gambling task similar to the reverse condition in the current study. In their study, both participants acted alternately as performers and observers.

Interestingly, female observers showed an FRN when their partners lost, i.e., even when this loss led to a gain for them. In contrast, male observers presented an FRN for gains of their partner which led to a loss for themselves. The authors discussed these results in terms of gender differences in empathy, suggesting that women's FRN was driven by an empathic response to the loss of their partner (overruling the response to their own gain), whereas men's FRN was mainly driven by the trial's outcome for themselves. We did not replicate these results in the current study, however, as in the reverse condition we found observer's FRN for the observer loss/performer gain trials. This is in line with the results found by Itagaki and Katayama [21] in which subjects were paired with a "virtual" performer in either a parallel or a reverse condition. In both conditions participants showed an FRN whenever they incurred a loss. A recent study by Leng and Zhou [22] is also important in this regard: Whereas the observation of a loss of a performer elicited an FRN in observers, its amplitude was not modulated by the personal relationship between the performer and observer, i.e. observers watching a close friend losing money and observers watching a complete stranger had indistinguishable FRNs. As one would expect a greater empathic response when watching close friends, Leng and Zhou's result argues against an empathic account. The fact that the FRN in the neutral and parallel conditions was of similar magnitude in the present condition also suggests that empathy might play little or no role in the generation of the FRN, as one would expect the FRN to be bigger in the parallel condition that features both, a consequence to oneself and to another person.

Altogether these results suggest that the FRN found for observers might be driven by two processes: one that evaluates the direct consequences to the observer and another that evaluates the consequences to others. The latter process governs the generation of the FRN, if observation of another person's actions has no consequences to the observer, whereas the former should be mainly responsible for the FRN seen in the parallel and reverse conditions. It might be explained similarly to the FRN in performers and the ERN-response in performance monitoring tasks. For theses situations, the reinforcement learning theory [23] holds that dopaminergic neurons in the ventral tegmental area projecting to frontal areas decrease their activity whenever an event has an outcome that is worse than expected. This decrease of dopaminergic input to the medial frontal cortex leads to a disinhibition of pyramidal neurons in this area which in turn leads to a mediofrontal negative response in the ERP (i.e., Error Related Negativity, ERN or Feedback Related Negativity). Similarly, observed losses in the parallel condition or gains of the performer in the reverse condition should produce a decrease in the dopaminergic activity in the observer which in turn triggers an FRN. A recent study by Yu and Zhou [24] has demonstrated that an FRN-like response can also be observed for missed wins in performers. In their experiment, participants had to decide whether or not they would bet on a given trial. For "no bet" trials feedback was provided regarding the potential outcome. Interestingly, in this condition potential (but not realized) wins were associated with a more negative mediofrontal response relative to potential losses. The authors speculated that this effect might be due to a "counterfactual comparison process" that treated the missed win as a negative feedback and the missed loss as a positive feedback in the "no bet" trials.

Interestingly, in our study, magnitude × valence interactions reflecting higher differences for the maximum magnitude trials were found in the parallel and reverse conditions but not in the neutral condition. This may reflect the greater impact of the gains and losses on the observer in the parallel and reverse conditions and provides further support for the two process account of the FRN in observers mentioned above. The second system that might be involved in the generation of the FRN in observers appears to be related to emotional/empathic aspects. This system might have been relevant in particular in the neutral condition. Empathy is a multifaceted concept that according to Decety and Jackson [25] entails three main components: an affective response to another's feelings, the cognitive capacity of perspective-taking and regulatory mechanisms that keep track of the origins of the feelings (self vs. other). Importantly, one influential model of empathy, the perception-action model [26], states that the perception of an emotional state in others automatically activates the corresponding representations of that emotion as well as associated somatic and autonomic responses in the observer. The theory is based on the more general idea that perception and action are represented in shared networks [3]. Recently, functional imaging studies could provide evidence for this theory by showing for instance that the observation of somebody in pain and pain perception activated overlapping brain regions [4,27]. To the degree that the FRN reflects or is modulated by an emotional evaluation of an outcome, the observer's FRN in the neutral condition might reflect the empathic response to the performer's loss.

There is some evidence that midfrontal negativities related to performance monitoring (ERN, FRN) are subject to emotional influences [28-32]. Luu and colleagues [31], for instance, could observe group differences in the ERN related to participants' trait anxiety. Moreover, recent studies demonstrated an effect of short-term emotions on the ERN such that the presentation of unpleasant IAPS pictures during a simple choice reaction time task led to an increased ERN [32], but see [33]. The present findings suggest that a participant's emotional response to loss of the performer might have induced an FRN even when there were no direct consequences for themselves. Interestingly, two recent functional Magnetic Resonance Imaging (fMRI) papers [34,35] have reported that observation of errors committed by others activates mediofrontal structures (anterior cingulate cortex and supplementary motor area), even when the error translated into a positive outcome for the observer. These findings are at odds with the current results, as in our experiment the observer's reaction to performer errors was modulated by the consequences of the errors for the observer. Please note, that the FRN has been tightly linked to activity of medial frontal cortex and thus, it is generally assumed that fMRI (mediofrontal activation) and EEG (FRN) should lead to comparable results. At present, we are unable to resolve the contradictions between the fMRI results [34,35] and the ERP results of the current paper.

Our suggestion of two mechanisms contributing to the observer's FRN (evaluative and empathic) can help to explain the differences between the results of Fukuhima and Hiraki [19] and the present findings. Whereas both processes might come into play when observing others' decisions and their consequences, they might be modulated by different factors. In particular, empathy has been shown to be modulated by gender [5,36], fairness [5] and emotional sensitivity towards others [4,5,37]. On the other hand, the evaluative component could be modulated by other factors, such as magnitude [38], probability [39] or amount of information provided by the feedback [40]. Importantly, in the reverse condition the two components would compete: whereas the empathic component would lead to an FRN in response to the other's loss, the evaluative component would trigger an FRN to one's own loss (i.e. the other's gain). The personal acquaintance of participants in the Fukuhima and Hiraki [19] study might have enhanced their empathic response, which could have overriden the evaluative component, at least in the female participants (see however Leng and Zhou [22]). However, in the present study participants did not know each other before the experiment, leading to a predominant response from the evaluative component.

## Conclusions

In conclusion, the present results show that the mere observation of other's losses elicits an FRN in the observer even without any direct engagement in the task or any relationship to the performer. In addition, our results in the three different conditions suggest that the observation of the performance of another person performing a task may activate two different evaluative processes in the brain, both modulating the FRN: one is driven by the outcome of the other person and may be related to empathy whereas the other evaluates the consequences for oneself.

## Methods

All procedures had been cleared by the local ethical review board.

### Subjects

Ninety-six right-handed healthy volunteers (50 women, age range 21-46 years old, mean age 23.7) participated in the study after giving their written consent. None of them had a history of neurological or psychiatric disorders. All subjects were paid 7 € per hour plus any rewards won in the task.

### Experimental design

Subjects participated in gender-matched pairs (always man-man or woman-woman) in the experiment. Care was taken that participants did not know each other before the experiment, or had any kind of relationship. At the beginning of the experiment, one participant was randomly selected to perform the task ("performer") and the other was instructed to observe the experiment ("observer"). The performer was instructed to play a monetary gambling task while the observer was told to observe the task and count certain events of the experiment (see below). Participants were seated in front of a screen in a sound-attenuating chamber.

The task used was a simplified version of the monetary gambling task [9,14,15]. Each trial began with a fixation cross that remained on the screen for two seconds and was then replaced by two numbers presented in white on black background. Only two possible displays were presented, either [25][5] or [5][25]. Performers had to make an obligatory button press response with their left or right index-finger, indicating the selected number. For example, in the [25][5] display a left button press would indicate the selection of the number 25, and a right button press the selection of the number 5. After the choice, the selected number turned blue to ensure that the observer was aware of the performer's choice. Finally, one second later, the result of the selection was shown. In 76% of the trials one number turned red while the other one turned green. If the number selected by the participant changed to red, this signalled a loss of the corresponding amount in Euro cent; a green number indicated a gain. Unlike the Gehring and Willoughby [9] task, our task did not feature trials in which both numbers turned green or red. In addition to the standard trials described above (76%), two further conditions were created to assess the brain's responses to unexpected rewards and losses: In 12% of trials (boost trials) one of the two choices turned into 125 (gain or loss) while the other number remained the same. In the remaining 12% of trials (similar trials), the selected number increased its value by 2 (25 turned to 27 and the 5 turned to 7, both in gains or losses). The rationale for including the boost trials was to study the impact of unexpectedly large gains or losses on brain activity, as under such conditions animal experiments have shown enhanced discharge of mesencephalic dopamine neurons. As the boost trials were both, infrequent and unexpectedly large, the similar trials were included that were infrequent but left the sums virtually unchanged. It turned out, however, that because of their lower number brain responses to these trial types had a rather low signal to noise ratio and a high interindividual variability. We will therefore focus on the standard trials in the remainder of the paper.

The experiment consisted of eight blocks, each comprising 112 trials. The outcomes were pseudorandomized with the constraint that each outcome condition had to occur with a certain frequency. The performer was initially endowed with the sum of 3 € and was instructed to try to gain as much as possible. After each block the performer's accumulated amount of Euros was shown on the screen. The observer did not have to make any selection but was instructed prior to each block to count the number of occurrences of a specified event in that block, i.e. number of high standard gains (+25), low standard gains (+5), high standard losses (-25) or low standard loses (-5). The instruction changed each block in order to keep the observer's attention. At the end of each block, the observer had to tell the experimenter the number of events he had counted, e.g., how often the performer lost 5 cent.

Three different conditions were introduced differing in how the gain of the observer was coupled to the choices of the performer: In the neutral condition, observers received 3 € on top of their hourly wage regardless of the performance of the "performer". In the parallel condition, observers won or lost the same amount of money as the performer. Finally, in the reverse condition, the observer won when the performer lost and vice-versa. Sixteen different observer/performer pairs participated per condition.

### EEG recording

EEG was recorded using tin electrodes mounted in an elastic cap and located at 29 standard positions (Fp1/2, Fz, F7/8, F3/4, Fc1/2 Fc5/6, Cz, C3/4, T7/8, Cp1/2, Cp5/6, Pz, P3/4, P7/P8, Po1/2, O1/2) in both, performer and observer. Vertical eye movements were monitored with an electrode at the infraorbital ridge of the right eye. Electrode impedances were kept below 5 kOhm throughout the whole experiment. EEG was rereferenced offline to the mean of the activity at the two mastoid processes. The electrophysiological signals were filtered with a bandpass of 0.01-50 Hz (half-amplitude cutoffs) and digitized at a rate of 250 Hz. Trials with amplitudes of more than ± 100 μV in EEG or EOG or with amplifier saturation were automatically rejected off-line.

ERPs time-locked to gains and losses were averaged for epochs of 700 ms starting 100 ms prior to the stimulus (baseline). Due to the low number of trials in the boost condition, we did not analyze boost trials and focussed on standard trials only. The possible differences were tested by using an ANOVA with valence (gain and loss) magnitude (maximum and minimum) and all 29 electrode locations as within-subject factors in a time window 40 ms around the peak of the gain minus loss difference at Cz.

## Authors' contributions

JMP, UMK and TFM codesigned the study. JMP, SS and AS performed the experiments and analyses. JMP, UMK and TFM wrote the paper. All authors read and approved the final manuscript.
